# New generation of wearable goniometers for motion capture systems

**DOI:** 10.1186/1743-0003-11-56

**Published:** 2014-04-11

**Authors:** Alessandro Tognetti, Federico Lorussi, Gabriele Dalle Mura, Nicola Carbonaro, Maria Pacelli, Rita Paradiso, Danilo De Rossi

**Affiliations:** 1 Research Center “E.Piaggio”, University of Pisa, Via Diotisalvi 2, Pisa, Italy; 2 Information Engineering Department, University of Pisa, Via Caruso 2, Pisa, Italy; 3 Smartex S.r.l., Via Giuntini 13L, 56023 Navacchio, Pisa, Italy

## Abstract

**Background:**

Monitoring joint angles through wearable systems enables human posture and gesture to be reconstructed as a support for physical rehabilitation both in clinics and at the patient’s home. A new generation of wearable goniometers based on knitted piezoresistive fabric (KPF) technology is presented.

**Methods:**

KPF single-and double-layer devices were designed and characterized under stretching and bending to work as strain sensors and goniometers. The theoretical working principle and the derived electromechanical model, previously proved for carbon elastomer sensors, were generalized to KPF. The devices were used to correlate angles and piezoresistive fabric behaviour, to highlight the differences in terms of performance between the single layer and the double layer sensors. A fast calibration procedure is also proposed.

**Results:**

The proposed device was tested both in static and dynamic conditions in comparison with standard electrogoniometers and inertial measurement units respectively. KPF goniometer capabilities in angle detection were experimentally proved and a discussion of the device measurement errors of is provided. The paper concludes with an analysis of sensor accuracy and hysteresis reduction in particular configurations.

**Conclusions:**

Double layer KPF goniometers showed a promising performance in terms of angle measurements both in quasi-static and dynamic working mode for velocities typical of human movement. A further approach consisting of a combination of multiple sensors to increase accuracy via sensor fusion technique has been presented.

## Background

Recently, a novel type of wearable sensor capable of detecting strain fields has been proposed [1-11]. Textile based deformation sensors can be produced by coating a thin layer of piezoresistive material on conventional fabrics [1,4,6] or by knitting conductive yarns with non-conductive yarns [2,3,7,9]. In other works, conductive threads are stitched [10,11] or attached [5,8] to the top of the fabric. The main features of textile deformation sensors are flexibility and the preservation of the mechanical properties of the garments on which they are applied. Textile deformation sensors have several advantages compared to solid-state sensors: negligible weight, thickness and possibility of spreading a high number of measuring points over a flexible substrate. Sensing garments can be designed by applying sensor strips to specific locations on normal cloth. Changes in body shape and/or geometry due to human movements can in principle be estimated by reading variations on the measured strain.

However, it may be difficult to recover the relationship between fabric strains and biomechanics parameters, such as tri-dimensional geometry or angles. Several solutions have been proposed to address this issue. In [12] a multivariate interpolation on a grid of sensors was used. Laviola in [13] reviewed the algorithms for hand posture recognition; Gibbs and Asada [5] described a knee-sensing garment made with conductive fibers attached to flexible skin-tight fabrics. Mattmann et al. [14] combined a supervised learning algorithm with conductive thread sensors for the detection of torso movements. In [15] coated sensing fabrics are obtained by the integration of conductive elastomer (CE) materials on textile fabrics by an ad hoc screen printing procedure with variable topology. An application of CE sensors aimed at detecting the upper limb movement for neurological application is described in [16-18]. Generally, CE based sensing garments perform well for slow and wide movements, while the accuracy, transient time and hysteresis has limited their use in reconstructing fast and small movements, such as anatomical torsion. In addition, retrieving subject posture from textile strain measurements is highly affected by the relative position of sensors with respect to the joint being monitored. This issue has been addressed with the use of tight-fitting garments, which can reduce user acceptance especially in home rehabilitation contexts. Even in the case of adherent garments, it is not easy to obtain reproducible results due to the inevitable sliding/bending of the sensors on the textile and to the difficulty of wearing the garment in the same way after donning and doffing. These latter aspects have limited joint angle tracking due to the necessity of using a complex and long lasting calibration procedure [6] or have restricted sensing garment usage in gesture classification applications [8].

In [19], CE sensors were configured in a double layer structure capable of direct angular measurements for application in rehabilitation and biomechanics. Since these double layer angular sensors are less sensitive to precise positioning and to their intermediate bending profile, they have the potential to solve some of the issues described for textile based strain transducers.

In [7,9,20], Knitted Piezoresistive Fabric (KPF) sensors were demonstrated as a good tool for biomechanical and cardiopulmonary data acquisition and constitute an improvement on CEs. Compared to CE, KPF materials perform better in terms of response time, making them more suitable for wearable motion-capture applications. In fact, the transient time of CE sensors is very long and requires dedicated algorithms for predicting the final output after solicitation [6,21]. In addition, producing CE sensors entails using trichloroethylene with consequent rigid constraints on manufacturing sensors. In [22], a data glove based on KPF used as strain sensors was developed and successfully tested in monitoring human hand gestures.

In this work we exploited KPF technology to create a new generation of wearable goniometers inspired by the methodology introduced in [19]. The use of a new material led to a new theoretical approach compared to previous works aimed at improving the performance of CE sensors.

## Methods

### Single and double layer angular sensor working principle

This section describes the basic theoretical aspects of textile-based angular sensors. Both single and double layer configurations were analyzed according to the guidelines described in [19] for conductive elastomer sensors and differences between them are highlighted.

**Figure 1:**
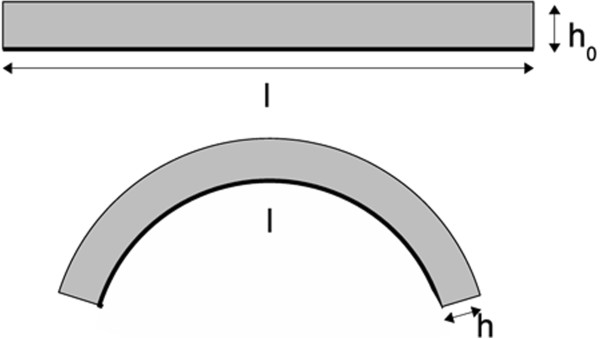
**Single layer CE sensor (light gray) attached to the inextensible substrate represented by the black line of length *l *.**

In [19] the working principle of a single layer CE angular sensor is described extensively. Single layer (*SL*) sensors are made of piezoresistive films (i.e. with a negligible thickness compared to length and width) attached to textile or other flexible substrates. The following assumptions form the basis of the analysis: (*i*) the sensing layer is characterised by isovolumetric deformations, (*ii*) the material resistivity *ρ* is constant, and (*iii*) the fabric/flexible substrate is not extensible under bending (i.e. one side of the film has a constant length *l* during bending). Figure 1 shows the CE film (in light gray) attached to the inextensible and flexible substrate represented by the thicker black line of length *l*. When the sample is flexed, the bottom side attached to the inextensible substrate maintains constant length *l*, while the sample height and length change according to its flexion.

Under these assumptions, we demonstrated that the layer electrical resistance *R*_*S* *L*_ of a specimen of length *l* parameterized in its arc length *s* is a function of the total curvature *Δα*, defined as the integral of the local curvature *k*(*s*) in the interval *s*∈ [ 0,*l*] [23] through the following relation:

(1) $$\begin{array}{ll} R_{\text{SL}} & =l\frac{\rho }{d\;h}-\frac{\rho }{d}\;\Delta \alpha +O\left(\underset{s\in (0,l)}{\sup}k{\left(s\right)}^{2}\right)\\ & =l^{2}\frac{\rho }{V_{0}}-\frac{\rho }{d}\;\Delta \alpha +O\left(\underset{s\in (0,l)}{\sup}k{\left(s\right)}^{2}\right) \end{array}$$

where *V*_0_ is the specimen volume, *d* its width, *Δα* is the angle between the tangent planes to the sensor extremities, *h*_0_ is the initial thickness of the specimen, and *O*(*sup*(*k*(*s*)^2^)) is a second-order infinitesimal function which tends to zero if the curvature *k*(*s*)→0. Note that *k*(*s*) assumes high values only when the sensor rapidly changes direction (as in cusps), which makes the function *O*(sup*s*∈(0,*l*)*k*(*s*)^2^) negligible in detecting human body shape. According to equation (1), the assumption of constant length guarantees the dependence of *R*_*S* *L*_ only on its bending. As underlined in [24], the dependence of *R*_*S* *L*_ on *Δα* is also not affected by the particular bending profile. By neglecting the second order error in *k*(*s*), the angle *Δα* can be computed by:

(2) $$\begin{array}{c} \Delta \alpha =\frac{d\;l^{2}}{V_{0}}-\frac{d}{\rho }R_{\text{SL}} \end{array}$$

**Figure 2:**
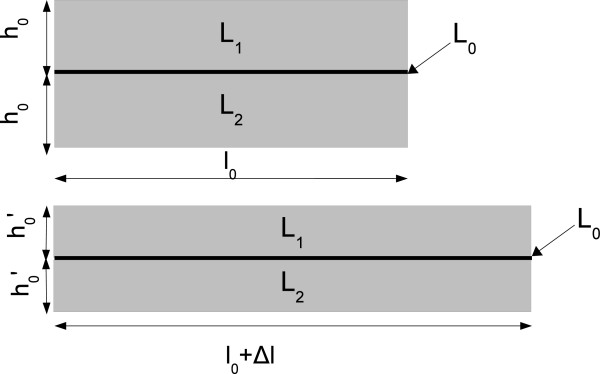
**Double layer angular sensor at rest (upper drawing) and stretched (lower drawing).** When the sensor is stretched, the thickness and length change their values ($h_{0}\to h_{0}^{\prime }$ and *l*_0_→*l*_0_+*Δl*).

Double layer (*DL*) angular sensors are based on the measurement of the resistance difference (*ΔR*_*D* *L*_=*R*_*L*1_−*R*_*L*2_) of two identical piezoresistive samples coupled via an insulating layer. This three-layer device is shown in Figure 2 where *L*_1_ and *L*_2_ are the piezoresistive layers with the same rest thicknesses *h*_0_. *R*_*L*1_ and *R*_*L*2_ are the electrical resistances of the two layers, respectively. *L*_0_ represents the insulating layer whose thickness is negligible compared to *h*_0_. *L*_0_ can be both stretched and bent.

**Figure 3:**
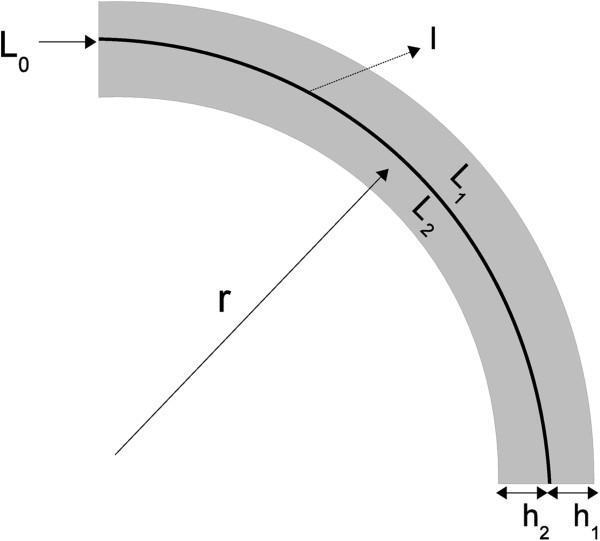
**Bent double layer sensor.***L*_1_ and *L*_2_ are the piezoresistive layers. *L*_0_ represents the insulating layer.

In the case of device deformation without bending, as represented at the bottom of Figure 2, the electrical resistance difference between the two layers remains zero, since both the sensing layers undergo the same transformation. When the device is flexed (as in Figure 3), [19] demonstrated that there is a linear dependence between *ΔR*_*D* *L*_ and *Δα*:

(3) $$\begin{array}{c} \Delta R_{\text{DL}}=R_{L1}-R_{L2}=2\frac{\rho }{d}\Delta \alpha +O\left(\underset{s\in (0,l)}{\sup}k{\left(s\right)}^{3}\right) \end{array}$$

This configuration proves that the error in angle estimation is reduced to a third order infinitesimal function with the maximum of the curvature *k*(*s*) [19]. While the hypothesis of iso-volumetric deformation and constant resistivity are still needed, the inextensibility can be removed since in (3) there is no dependence on the actual sample length. By neglecting the third-order error term, the angle *Δα* can be estimated by:

(4) $$\Delta \alpha =\frac{d}{2\rho }\Delta R_{\text{DL}}$$

### Sensor development

Textile-based sensors were produced using knitted piezoresistive fabrics (KPF) which contain 75% electro-conductive yarn (Belltron®;, produced by Kanebo Ltd) and 25% Lycra®;, manufactured as single jersey in a circular machine as described in previous works [7,25]. Circular electronic seamless-wear knitting machines by Santoni ^*T* *M*^[26] were used to produce piezoresistive fabrics due to their capability to handle yarns with high elastic recovery. Flat knitting machines are more sophisticate in term of stitch selections, but less efficient in the handling of elastic yarns and in the production time. Moreover a Santoni machinery requires only the use of 4/8 spools of yarns compared with warp knitting machines. A conductive bi-component fiber yarn based on polyamide loaded with carbon particles is used in combination with lycra to make this sensor. Piezoresistive fabric sensors change the electrical resistance according to the strain; the variation in electrical properties is due to the modification of the interconnections geometry inside the fabric structure. Usually this property can be observed in stretchable fabrics where the elongation of the fibers affects the flow of carrier inside the structure. When the conductivity of the yarn is due conductive particles as in bi-component fibers, the elongation of the yarn affects the charge transport mechanisms. The interconnections between the fibers and stitches are altered by the applied deformation. The elongation of the fabric modifies the distance between stitches as well as the arrangement of the fibers in the yarn leading to a different geometry of interconnections.

**Figure 4:**
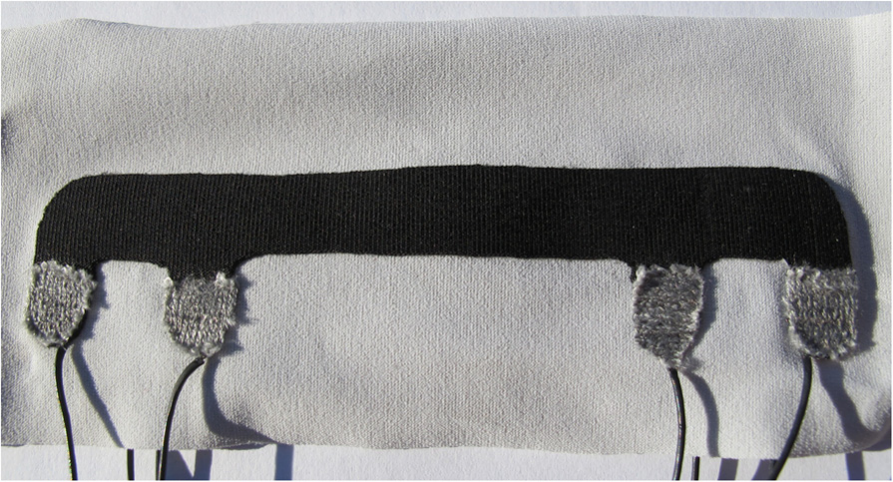
Detail of one side of a double layer KPF sensor focusing on the connections between sensor and conductive wires.

The samples were created according to the single and double layer design introduced in the previous section and hereinafter indicated as SL and DL sensors, respectively. The SL structure is made by coupling one rectangular KPF sample with an elastic fabric through a double-sided adhesive membrane. The DL structure is produced by adding another identical KPF layer to the back of the elastic fabric using the same adhesive membrane of the SL. Each piezoresistive layer has four semicircular pads specifically designed for sensor wiring. Figure 4 shows the electrical connections made of textile conductive yarns with PVC insulation (Bekinox®;, produced by Bekaert). After removing the insulating material in the thread extremities, the connections were fixed using an ultrasonic welding device.

**Figure 5:**
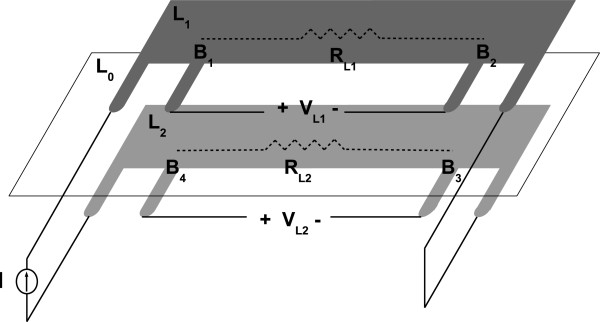
**Functional structure of a double layer KPF sensor.** The central pads are for voltage acquisition, while the extremities are used for current supply and for the connection of the two single layer sensors.

**Figure 6:**
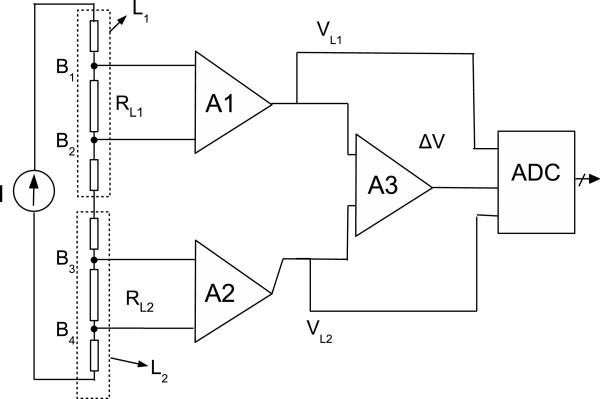
**KPF goniometer electrical equivalent and block diagram of the electronic acquisition system.** The high input impedance stage is based on two instrumentation amplifiers (A1 and A2). A3 is a differential amplifier. The voltage output *ΔV* is proportional to the resistance difference and thus to the flexion angle.

Figure 5 shows the structure of the DL sensor, where an insulating layer *L*_0_ is placed between the two piezoresistive layers *L*_1_ and *L*_2_. The topology of the connecting pads was specifically designed for a four-point measurement in order to minimize the effect of contact resistances. A constant current *I* is supplied through the external pads and the voltages $V_{L1}=V_{B_{1}B_{2}}$ and $V_{L2}=V_{B_{4}B_{3}}$ (Figures 5 and 6) between the internal pads are measured and can be related to resistances *R*_*L*1_ and *R*_*L*2_ (by dividing by *I*). As the curvature angle is related to the difference between resistances *R*_*L*1_ and *R*_*L*2_, a dedicated electronic was designed to continuously acquire *ΔR*_*D* *L*_.

The custom-designed measurement setup is shown in Figure 6. A current generator supplies the two series of impedances with a constant current (*I*) and the acquisition system is a high input impedance stage constituted by two instrumentation amplifiers (*A*1 and *A*2). The outputs are amplified and the difference is measured in the following stage (*A*3). Both the resistance difference and the single layer resistance values are then acquired by a National Instruments acquisition board. The goniometers (Figure 4) that we tested have an overall rest length of 100 *mm*, which corresponds to the distance between the current carrying pads. The rest distances between the voltage sensing pads *B*_1_*B*_2_ and *B*_4_*B*_3_ are 50 *mm*. The rest width and thickness are 10 *mm* and 0.5 *mm*, respectively.

### Double layer device calibration

Equation (3) highlights the advantages of independence on the length variation of the device and the reduced errors in approximating the device’s electrical resistance via a Taylor expansion. On the other hand, it only holds if the two piezoresistive layers have the same electrical properties. This property can be difficult to obtain from a practical point of view and the advantage of coupling two layers may be lost. To compensate for differences between the two piezoresistive layers, a calibration procedure can be applied. Considering the equation (1) for the layers *L*_1_ and *L*_2_, truncated at the second order term, as in the following:

(5) $$R_{L_{1}}=l^{2}\frac{\rho }{V_{0}}-\frac{\rho }{d}\;\Delta \alpha$$

and

(6) $$R_{L_{2}}={\left(l^{\prime }\right)}^{2}\frac{\rho }{V_{0}^{\prime }}+\frac{\rho }{d^{\prime }}\;\Delta \alpha =q_{0}+l^{2}\frac{\rho }{V_{0}}q_{1}-\frac{\rho }{d}\;\Delta \alpha q_{2}$$

where *q*_0_, *q*_1_ and *q*_2_ are three corrective coefficients which take into account the geometrical and electrical diversity between the two layers due to the production process. *q*_0_, *q*_1_ and *q*_2_ are invariant with respect to the curvature and can be estimated by comparing the electromechanical characteristics of the two sensing specimens which will be introduced below. By multiplying equation (5) by *q*_2_, subtracting (6) and solving with respect to *Δα* and *l*, the following expressions are obtained:

(7) $$\Delta \alpha =\frac{q_{1}R_{L1}-R_{L2}+q_{0}}{\frac{\rho }{d}(q_{1}+q_{2})}$$

and

(8) $$l=\sqrt{V_{0}\frac{q_{2}R_{L1}+R_{L2}-q_{0}}{\frac{\rho }{d}(q_{1}+q_{2})}}$$

### Experimental set-up

KPF samples were characterised in quasi-static conditions for elongation and flexion both for SL and DL configurations through dedicated bench testing setups. In addition, DL dynamic performance in terms of flexion were preliminary evaluated on a human subject in comparison with inertial measurement units (IMUs).

#### Quasi-static elongation test

**Figure 7:**
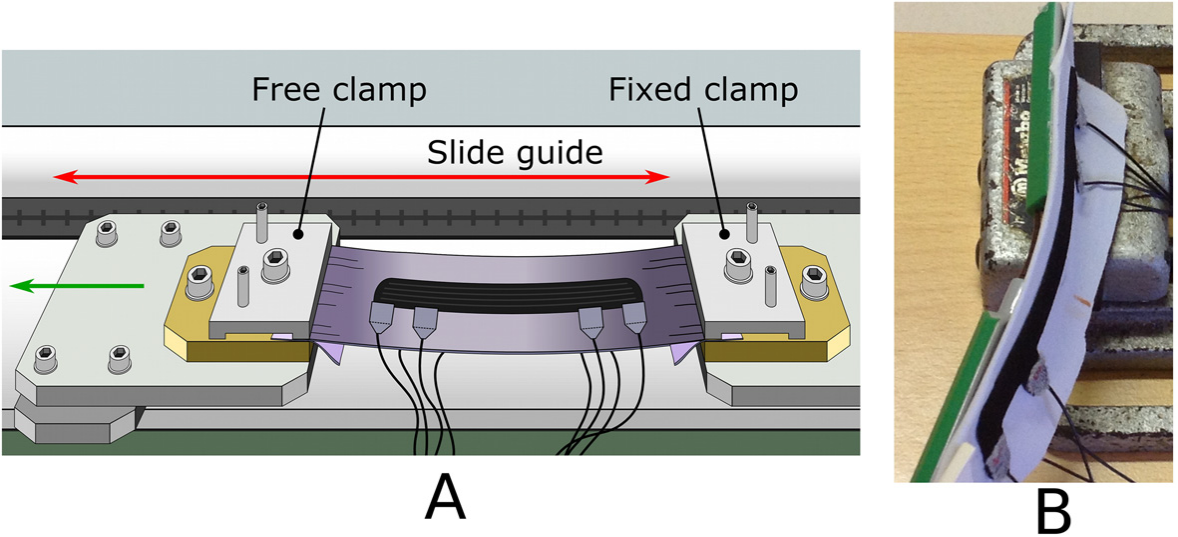
**Quasi-static test set-up.** Figure 7A shows the quasi-static elongation test set-up. The sample is attached to two clamps, one fixed (on the right) and the other one free to move (on the left). The slide has an 11 cm maximum range. The tested sample was subjected to a total deformation of 5 *mm* through 11 equivalent steps, both in elongation and shortening. Figure 7B shows the experimental setup for flexion characterization. The sensor is attached to a flexible substrate and is coupled to a commercial electrogoniometer. One extremity of the structure was clamped in a bench vice and the other one remained free to pivot around.

Elongation response was assessed using a custom-designed electro-dynamic testing system based on a linear motor controlled by a PLC and able to apply strain cycles with controlled amplitude and velocity. Each layer resistance (*R*_*L*1_, *R*_*L*2_) and their difference (*ΔR*_*D* *L*_) were recorded as a function of the applied elongation. As shown in Figure 7(A), one extremity of the sample was attached to a fixed point while the other extremity was free to move. The sample was subjected to a total deformation of 5 *mm* through 10 equivalent steps, both in terms of elongation and shortening. Each step lasted one minute and the average value of the recorded data was computed by the last 30 seconds of the corresponding step.

#### Quasi-static flexion test

Quasi-static flexion characterization was carried out by relating *R*_*L*1_, *R*_*L*2_ and *ΔR*_*D* *L*_ with the output of a commercial electrogoniometer. Electrogoniometers are commonly used as a gold standard for angle measuring in biomechanical applications [27]. The KPF sensor was attached to a flexible substrate composed of woven fiberglass cloth and epoxy resin (i.e. standard printed circuit board material). Then, a two-axis electro-goniometer SG110 by Biometrics (±2°*C* accuracy) was attached to the opposite side of the flexible substrate. One extremity of this structure was clamped in a bench vice and the other remained free to pivot around, Figure 7(B). Starting from 0° the structure was bent to 90° through 13 steps. In each step, the sensor was held to rest for about 60 seconds and the average value of the recorded data was computed within the last 30 seconds. The test was performed both in flexion (from 0° to 90°) and extension (from 90° to 0°).

#### Dynamic test

**Figure 8:**
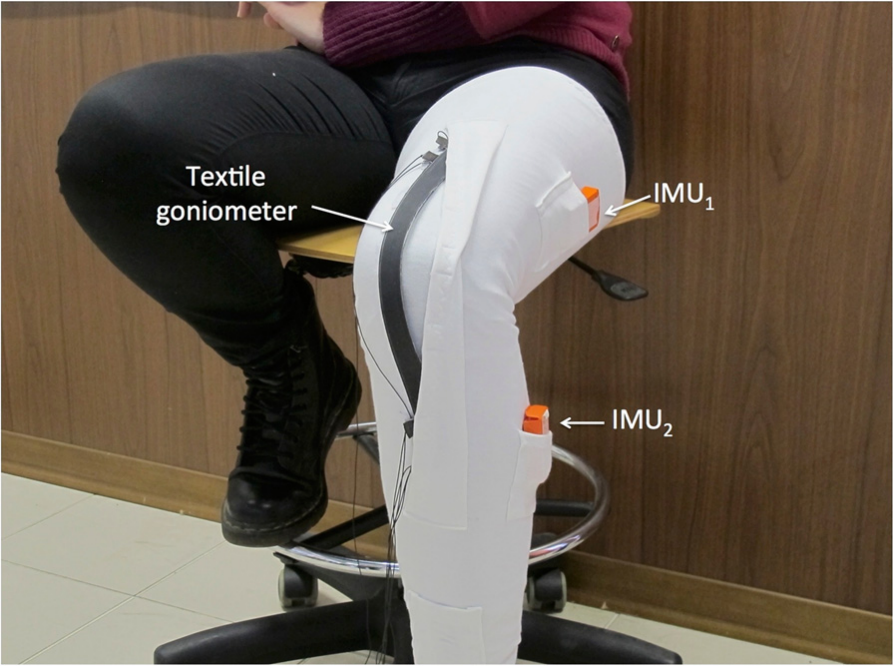
Dynamic test set-up: double layer KPF goniometer applied to a band for the detection of knee flexion/extension; two IMUs are fixed to the thigh and on the calf in order to provide a reference measurement of the knee flexion/extension angle.

For a preliminary evaluation of the DL sensor performance in dynamic conditions, a double layer KPF sensor was applied to a knee band and compared with the outputs of two IMUs (MTw provided by XSENS [28]) placed on the thigh and on the calf, and used as a gold standard measurement instrumentation. In this case, a goniometer, longer than the one used in the quasi-static tests described above (40 cm, distance between the internal pads 30 cm), was used to entirely cover the knee joint. A representation of the set-up is shown in Figure 8. Several tests were performed by moving the knee in controlateral monopodalic standing at different velocities (slow, medium and fast).

### Data analysis

Stretching and bending data, acquired on SL and DL sensors using the setups described in the previous section, were analysed in order to assess the performance of: 

• SL in stretching (i.e. *R*_*S* *L*_ variation with respect to the applied strain)

• SL in bending and estimate the error by applying the relationships (1) truncate at the second order term in *Δα*

• DL in stretching and estimate the error by supposing that both layers have identical behaviour

• DL in bending and estimate the error by applying the relationships (3) trunked at the third order term in *Δα*

and in addition to: 

• Estimate the parameters *q*_0_, *q*_1_ and *q*_2_ introduced in (6) to calibrate the double layer device

• Preliminarily assess dynamic performance of DL in bending

Hereinafter, to simplify the notation, *R* denotes the resistance of a single layer device or of one of the two layers (instead of *R*_*S* *L*_), while *ΔR* indicates the resistance difference between the two layers (instead of *ΔR*_*D* *L*_)

#### Single layer resistance vs. stretching characteristics

A single layer piezoresistive device was tested to estimate the linearity of the electromechanical characteristic and evaluate the errors introduced by hysteretic phenomena. Ten elongation/shortening cycles were performed with a total elongation of 5 *mm* by steps of 0.5 *mm*. The average values and the standard deviations of the electrical resistance were calculated (equations 9 and 10 respectively) for each imposed deformation *d*_*i*_ over the set of cycles.

(9) $${\bar{R}}_{d_{i}}=\frac{1}{P}\underset{p}{\sum }R_{d_{i},p}$$

(10) $$\sigma_{R_{d_{i}}}=\sqrt{\underset{p}{\sum }\frac{1}{P}{\left(R_{d_{i},p}-{\bar{R}}_{d_{i}}\right)}^{2}}$$

In (9) and (10) *p* denotes the *p*^*t* *h*^ trial executed for a certain deformation *d*_*i*_ and *P* the total number of trials. To roughly estimate the SL electromechanical properties, ${\bar{R}}_{d_{i}}$ vs. *d*_*i*_ characteristic was approximated by a linear function and the deformation sensitivity (*S*_*d*_) was computed as the slope of its linear approximation.

#### Using the single layer device as a goniometer

Relationship (1) truncated at the first order term relates the angle *Δα* to the electrical resistance *R*. If the length *l* of one side of the device is maintained constant by an external constraint (e.g. the layer is glued on an inextensible film), the bending angle *Δα* can be estimated using relation (2). In order to validate (2), fifteen flexion (increasing angles) and extension (decreasing angles) cycles were applied. In each cycle the electrical resistance was acquired for 13 different angles *Δα*_*i*_ in the 0° – 90° range using the set-up described above. The average values ${\bar{R}}_{\Delta \alpha_{i}}$ and the standard deviations $\sigma_{\Delta \alpha_{i}}$ of the electrical resistance were computed for each angular value:

(11) $${\bar{R}}_{\Delta \alpha_{i}}=\frac{1}{K}\underset{k}{\sum }R_{\Delta \alpha_{i},k}$$

(12) $$\sigma_{R_{\Delta \alpha_{i}}}=\sqrt{\underset{k}{\sum }\frac{1}{K}{\left(R_{\Delta \alpha_{i},k}-{\bar{R}}_{\Delta \alpha_{i}}\right)}^{2}}$$

where *k* is *k*^*t* *h*^ trial executed for a certain angle *Δα*_*i*_ and *K* the total number of trials.

${\bar{R}}_{\Delta \alpha_{i}}$ trend was approximated by a linear regression in the least square sense and the linear regression slope, i.e. the angular sensitivity of the single layer sensor *S*_*Δ* *α* *S* *L*_, was computed.

#### Using the double layer device as a goniometer

The double layer device was tested in stretching to prove *ΔR* independence on elongation and in bending to verify the relationship between angles and resistance differences. Elongation/shortening and flexion/extension cycles were the same as described for the single layer sensor.

Data collected in the elongation/shortening trials were used to calculate mean (13) and standard deviation (14) of *ΔR* as a function of the deformation applied, without using the calibration procedure described in the corresponding section.

(13) $${\bar{\mathrm{\Delta R}}}_{d_{i}}=\frac{1}{P}\underset{p}{\sum }\Delta R_{d_{i},p}$$

(14) $$\sigma_{\Delta R_{d_{i}}}=\sqrt{\underset{p}{\sum }\frac{1}{P}{\left(\Delta R_{d_{i},p}-{\bar{\mathrm{\Delta R}}}_{d_{i}}\right)}^{2}}$$

Flexion/extension data were computed to determine the relation between the calibrated sensor output *ΔR*^∗^ and the angle *Δα*. The calibrated sensor output (*ΔR*^∗^) can be retrieved from equation (7):

(15) $$\Delta R^{*}=q_{1}R_{L1}-R_{L2}-q_{0}.$$

Also in this case the mean (16) and the standard deviation (17) of *ΔR*^∗^ were calculated for each angular position, and the angular sensitivity of the double layer sensor *S*_*Δ* *α* *D* *L*_ was computed as the slope of the linear approximation of the ${\bar{\Delta R^{*}}}_{\Delta \alpha_{i}}$ trend.

(16) $${\bar{\Delta R^{*}}}_{\Delta \alpha_{i}}=\frac{1}{K}\underset{k}{\sum }\Delta R_{\Delta \alpha_{i},k}^{*}$$

(17) $$\sigma_{\Delta R_{\Delta \alpha_{i}}^{*}}=\sqrt{\underset{k}{\sum }\frac{1}{K}{\left(\Delta R_{\Delta \alpha_{i},k}^{*}-{\bar{\Delta R^{*}}}_{\Delta \alpha_{i}}\right)}^{2}}$$

#### Parameters identification and calibration

Relation (7) can be used both for calibrating the double layer device and for identifying the system parameters which are still unknown. The parameters involved in the reconstruction of the angle starting from the two resistance values are the sensor width *d*, its resistivity *ρ* and the dissimilarities between the two layers, which are expressed by *q*_0_, *q*_1_ and *q*_2_. These five variables can be summarized in a restricted set as:

(18) $$\left\{\begin{array}{c} a_{1}=\frac{q_{1}\;d}{\rho (q_{1}+q_{2})}\\ a_{2}=-\frac{d}{\rho (q_{1}+q_{2})}\\ a_{3}=\frac{q_{0}\;d}{\rho (q_{1}+q_{2})} \end{array}\right)$$

which transform the relationship (7) into:

(19) $$\Delta \alpha =a_{1}\;R_{L1}+a_{2}\;R_{L2}+a_{3}$$

In order to determine the vector (*a*_1_*a*_2_*a*_3_)^*T*^, a set of experimental trials were performed by imposing different angles on the device in Figure 4. After executing *m* measurements (corresponding to angles *Δα*_*i*_, *i*=0..*m*), the data collected were included in the linear system:

(20) $$\left[\begin{array}{c} \Delta \alpha_{1}\\ \Delta \alpha_{2}\\ \vdots \\ \Delta \alpha_{m} \end{array}\right]=\left[\begin{array}{ccc} R_{L1_{1}} & R_{L2_{1}} & 1\\ R_{L1_{2}} & R_{L2_{2}} & 1\\ \vdots & \vdots & \vdots \\ R_{L1_{m}} & R_{L2_{m}} & 1 \end{array}\right]\;\left[\begin{array}{c} a_{1}\\ a_{2}\\ a_{3} \end{array}\right].$$

If matrix *A* is defined as

(21) $$A=\left[\begin{array}{ccc} R_{L1_{1}} & R_{L2_{1}} & 1\\ R_{L1_{2}} & R_{L2_{2}} & 1\\ \vdots & \vdots & \vdots \\ R_{L1_{m}} & R_{L2_{m}} & 1 \end{array}\right],$$

the parameter values can be computed by

(22) $$\left[\begin{array}{c} a_{1}\\ a_{2}\\ a_{3} \end{array}\right]={\left(AA^{T}\right)}^{-1}A^{T}\left[\begin{array}{c} \Delta \alpha_{1}\\ \Delta \alpha_{2}\\ \vdots \\ \Delta \alpha_{m} \end{array}\right]$$

The relationship (22) was computed by considering a complete increasing/decreasing cycle corresponding to 26 angular measurements in the 0° – 90° range, performed with the set-up for quasi-static flexion characterization described in Figure 7b. The identification procedure we are describing holds for all the goniometers we tested. In the following, since numerical considerations are necessary to evaluate the adopted method, we consider the behaviour of a particular device. The values of the parameters for the chosen goniometer are:

(23) $$\begin{array}{ccc} a_{1}=1.3\;10^{-3} & a_{2}=-3\;10^{-4} & a_{3}=-58.7164 \end{array}$$

There is a notable difference between |*a*_1_| and |*a*_2_|, despite the fact that they are coefficients of the resistance of the two layers *L*_1_ and *L*_2_ whose geometrical properties are very similar. This may be caused by the numerical instability of the method used. In order to verify this, the conditioning number of *A*^*T*^*A* was computed:

(24) $$\mu_{26}(A)=∥A^{T}A{∥}_{2}∥{\left(A^{T}A\right)}^{-1}{∥}_{2}=7.5169\;10^{13}$$

and indicates that the matrix *A* is very unstable. The poor conditioning of the matrix can be caused by small angles between different rows (considered as vectors) or large differences in the norm of different columns. In order to prevent the small angles problem, selecting those related to very different values can reduce the number of equations. The parameters were identified by recomputing equation (22) on a set of three measurements corresponding to *Δα*=0°, 45°, 90°:

(25) $$\begin{array}{ccc} a_{1}=1.3\;10^{-3} & a_{2}=-3\;10^{-4} & a_{3}=-78.5450 \end{array}$$

(26) $$\mu_{3}(A)=1.0792\;10^{14}.$$

The conditioning number gets worse, thus the second strategy, consisting in acting on the columns of *A*, is needed.

Note that *a*_3_ in *A* (*a*_1_*a*_2_*a*_3_)^*T*^ has the same value in each row and is a function of *q*_0_ through equation (18). As shown in the Results section, since *q*_0_ is strictly related to the average value of *ΔR* for the unbent sensor, *a*_3_ can be estimated separately. If the third coefficient is obtained independently by evaluating the average *ΔR* value in a short time from a static trial corresponding to *Δα*=0° the system (20) is reduced to

(27) $$\left[\begin{array}{c} \Delta \alpha_{1}\\ \Delta \alpha_{2}\\ \vdots \\ \Delta \alpha_{m} \end{array}\right]=\left[\begin{array}{cc} R_{L1_{1}} & R_{L2_{1}}\\ R_{L1_{2}} & R_{L2_{2}}\\ \vdots & \vdots \\ R_{L1_{m}} & R_{L2_{m}} \end{array}\right]\left[\begin{array}{c} a_{1}\\ a_{2} \end{array}\right]+\left[\begin{array}{c} a_{3}\\ a_{3}\\ \vdots \\ a_{3} \end{array}\right]$$

consequently:

(28) $$\left[\begin{array}{c} a_{1}\\ a_{2} \end{array}\right]={\left(B^{T}B^{T}\right)}^{-1}B^{T}\left[\left[\begin{array}{c} \Delta \alpha_{1}\\ \Delta \alpha_{2}\\ \vdots \\ \Delta \alpha_{m} \end{array}\right]-\left[\begin{array}{c} a_{3}\\ a_{3}\\ \vdots \\ a_{3} \end{array}\right]\right].$$

The numerical stability of the method, computed in the previous three angles, improves notably, as proved by

(29) $$\mu_{3}(B)=∥B^{T}B{∥}_{2}∥{\left(B^{T}B\right)}^{-1}{∥}_{2}=67.5488$$

and the computed values hold

(30) $$\begin{array}{cc} a_{1}=1.059\;10^{-3} & a_{2}=-1.067\;10^{-3}. \end{array}$$

The same coefficients, computed by considering only two measurements (*Δα*=0°, 90°), are

(31) $$\begin{array}{cc} a_{1}=1.065\;10^{-3} & a_{2}=-1.063\;10^{-3}. \end{array}$$

with a conditioning number

(32) $$\mu_{2}(B)=45.9070$$

Numerical output (30) and (31) are characterized by the same values (they differ in non-significant digits) with very similar conditioning numbers.

#### Dynamic test

The device in Figure 8 was calibrated according to the procedure described for the calculation of parameters *a*_1_ and *a*_2_. A zero-value was acquired when the knee was completely extended. IMUs were calibrated in the standard way, to virtually obtain the same orientation of the related frames in the same joint position. The flexion-extension angle deriving from IMU outputs was computed by taking into account the components of the rotation matrix which describe the orientation of the IMU frame on the calf with respect to the frame of the IMU placed on the thigh. The knee flexion-extension measured with the KPF goniometer *θ*_*g*_(*t*) was compared with the angle obtained by the two IMUs *θ*_*I* *M* *U*_(*t*). In addition, the two statistics of (33) and (34) were used to perform an inferential comparison between the measurement systems

(33) $$X=\bar{\theta_{\text{IMU}}-\theta_{g}}=\frac{1}{t_{1}-t_{0}}\int_{t_{0}}^{t_{1}}\theta_{\text{IMU}}(t)-\theta_{g}(t)\text{dt},$$

(34) $$\sigma =∥\theta_{\text{IMU}}-\theta_{g}{∥}_{2}=\frac{1}{t_{1}-t_{0}}\sqrt{\int_{t_{0}}^{t_{1}}{\left(\theta_{\text{IMU}}(t)-\theta_{g}(t)\right)}^{2}\text{dt}}.$$

A set of ten trials on different subjects was performed in three different conditions: slow-speed, medium-speed and high-speed flexions. For each trial, the statistics (33) and (34) were computed. A Student’s t-test was then performed to prove that the measurement samples obtained by the two different systems belonged to the same population (*H*_0_-hypothesis, i.e. X is a zero-mean random variable).

## Results

### Single layer resistance vs. stretching characteristics

**Figure 9:**
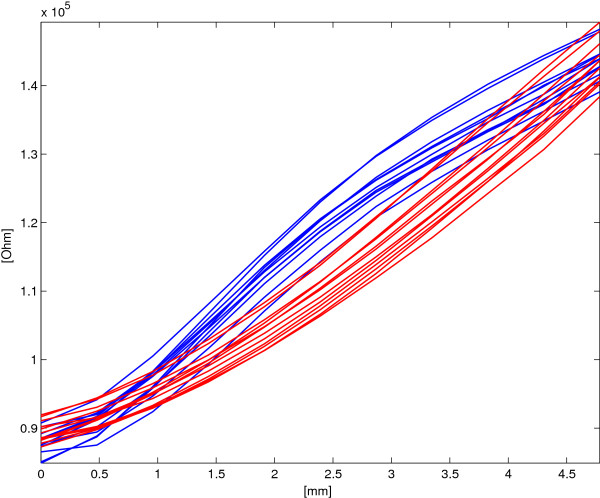
**SL sensor:** ***R* vs. applied deformation for both elongation (blue lines) and shortening (red lines).**

**Figure 10:**
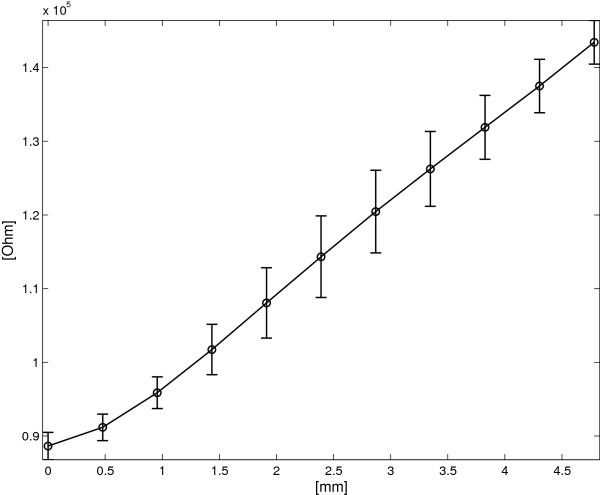
**SL sensor: average *R * vs. applied deformation for both increasing and decreasing deformation cycles.** The vertical bars represent two standard deviation units in length.

Figure 9 shows the relation between the SL electrical resistance and the deformation applied. The average *R* values and the corresponding standard deviations *σ*_*R*_ are reported in Figure 10 as a function of the deformation applied *d*. The deformation sensitivity *S*_*d*_ is 11950 *Ωmm*^−1^ and the maximum standard deviation $\sigma_{\text{Max}}=\underset{d_{i}}{\max}\sigma_{R_{d_{i}}}$ holds 5603 *Ω* for 2.8 *mm*, corresponding to an estimated standard deviation in terms of length of 0.4 *mm* which is comparable with the amplitude of the steps applied.

### Using the single layer device as a goniometer

**Figure 11:**
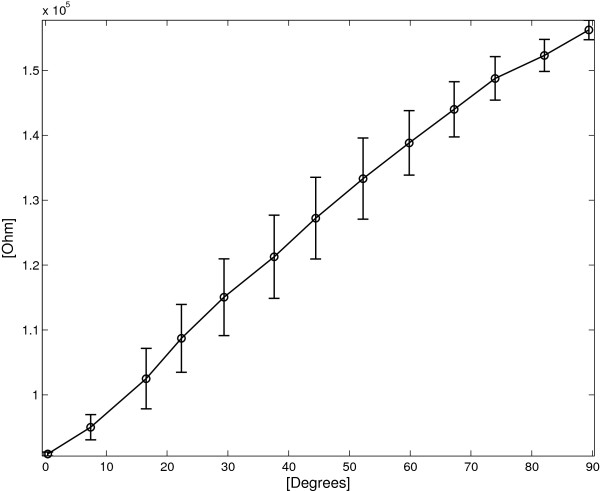
**SL sensor: average *R * vs. angle for both flexion and extension cycles.** The vertical bars represent two standard deviation units in length.

Figure 11 shows the angle/resistance characteristic of the SL device and the corresponding standard deviations. The angular sensitivity *S*_*Δ* *α* *S* *L*_ holds 763 *Ω*/° and the maximum standard deviation $\sigma_{\text{Max}}=\underset{\Delta \alpha_{i}}{\max}\sigma_{R_{\Delta \alpha_{i}}}$ holds 6405 *Ω* for *Δα*=37°, corresponding to an estimated angular standard deviation of 8.3°.

### Using the double layer device as a goniometer

**Figure 12:**
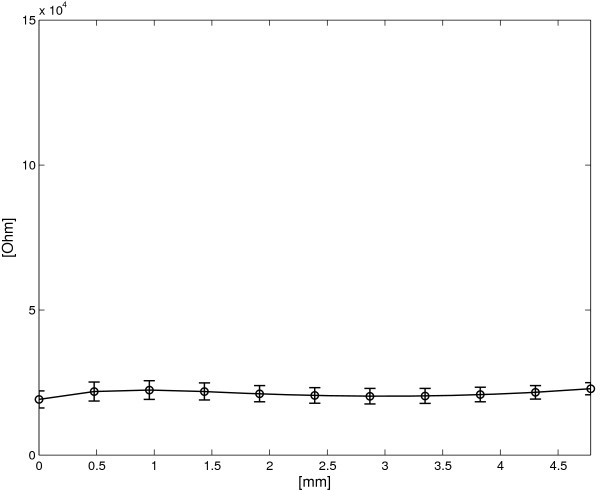
**DL sensor: average *Δ R * vs. applied deformation for both increasing and decreasing deformation cycles.** The vertical bars represent two standard deviation units in length.

Figure 12 shows the relation between the resistance difference of the double layer device and the applied deformation.

Note that the baseline of Figure 12 represents the dissimilarity between the behaviour of the two layers in terms of strain. As can be observed from the quasi-constant behaviour of the curve in Figure 12, *ΔR* is not dependent on *l* (i.e. *q*_1_≈1). For a first approximation, the average value of the function plotted in Figure 12 holds *q*_0_ and can be analogically subtracted by the signal deriving from one of the two layers or from their difference. Considering the acquisition diagram shown in Figure 6, *q*_0_ can be analogically subtracted by measuring the initial offset of the un-bended sensor, which is done by regulating the offset of one of the three amplifiers (*A*1, *A*2 or *A*3). After offset compensation, the relation between the angle *Δα* and the resistance difference *ΔR* can be obtained starting from equation (7) and (15) by imposing *q*_1_=1

(35) $$\Delta R^{*}=\mathrm{\Delta R}-q_{0}=\left(\frac{2\;\rho }{d}+q_{2}\right)\Delta \alpha$$

**Figure 13:**
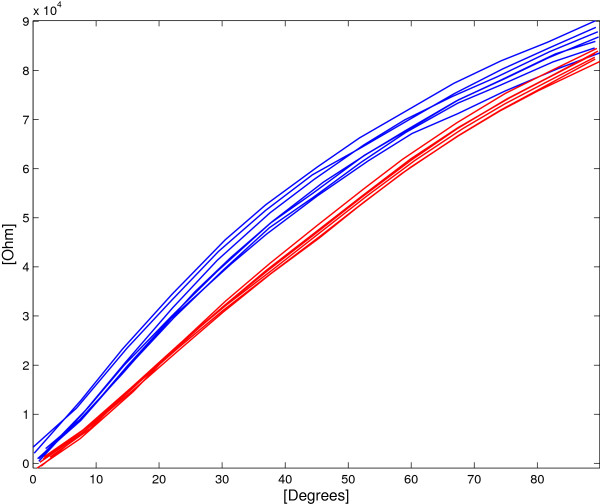
**DL sensor: *Δ R***^**∗**^** vs. *Δα * for increasing (blue lines) and decreasing (red lines) angles.**

Relation (35) was experimentally verified to evaluate the error in angular measurement. Figure 13 reports the data obtained in 15 trials for 13 different angles from 0° to 90°, acquired both in increasing and in decreasing measurements.

**Figure 14:**
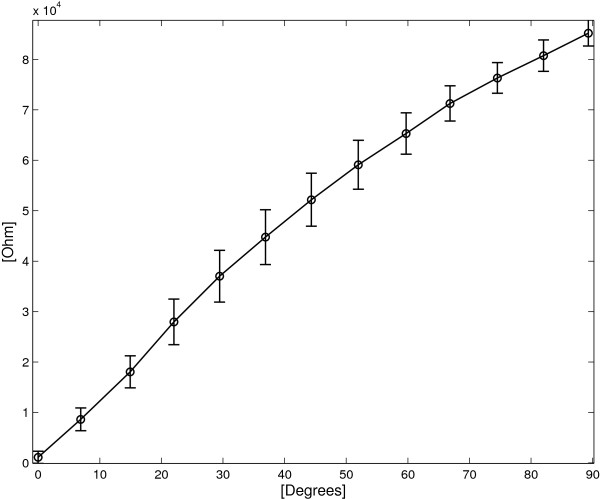
**DL sensor: average *Δ R***^**∗**^** vs. angle for both flexion and extension cycles.** The vertical bars represent two standard deviation units in length.

Figure 14 shows average *ΔR*^∗^ and the associated standard deviation plotted as a function of the the angle *Δα*. The angular sensitivity of the double layer sensor *S*_*Δ* *α* *D* *L*_ is estimated as 955 *Ω*/° and the maximum standard deviation *σ*_*M* *a* *x*_ was evaluated 5100 *Ω* for *Δα*=37°, corresponding to an angular error of 5.3°.

**Figure 15:**
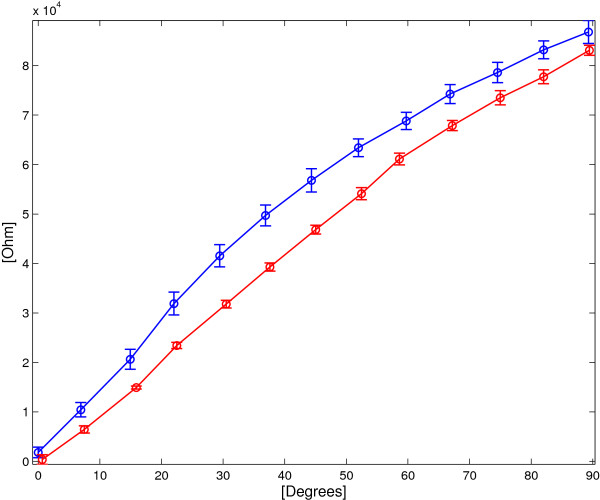
**DL sensor: average *Δ R***^**∗**^** vs. angle for flexion (red curve) and extension (blue curve) cycles.** The vertical bars represent two standard deviation units in length.

A further analysis was performed starting from the observation of the different sensor behaviour between flexion and extension (Figure 13). The mean and standard deviations of *ΔR*^∗^ were evaluated for increasing and decreasing angles separately (${\bar{\Delta R^{*}}}_{\Delta \alpha_{i}}^{I}$, $\sigma_{{\Delta R^{*}}_{\Delta \alpha_{i}}}^{I}$ and ${\bar{\Delta R^{*}}}_{\Delta \alpha_{i}}^{D}$, $\sigma_{{\Delta R^{*}}_{\Delta \alpha_{i}}}^{D}$ respectively) and plotted in Figure 15.

(36) $${\bar{\Delta R^{*}}}_{\Delta \alpha_{i}}^{I/D}=\frac{1}{K}\underset{k}{\sum }{\Delta R^{*}}_{\Delta \alpha_{i},k}^{I/D}$$

(37) $$\sigma_{{\Delta R^{*}}_{\Delta \alpha_{i}}}^{I/D}=\sqrt{\underset{k}{\sum }\frac{1}{K}{\left({\Delta R^{*}}_{\Delta \alpha_{i},k}^{I/D}-{\bar{\Delta R^{*}}}_{\Delta \alpha_{i}}^{I/D}\right)}^{2}}$$

The maximum standard deviations of the two characteristics were computed:

(38) $$\sigma_{\text{Max}}^{I}=\underset{\Delta \alpha_{i}}{\max}\sigma_{{\Delta R^{*}}_{\Delta \alpha_{i}}}^{I}=2.5\;\mathrm{K\Omega}$$

(39) $$\sigma_{\text{Max}}^{D}=\underset{\Delta \alpha_{i}}{\max}\sigma_{{\Delta R^{*}}_{\Delta \alpha_{i}}}^{D}=1.4\;\mathrm{K\Omega}$$

which corresponds to the estimated angular standard deviations of 2.4° and 1.48° respectively.

### Parameter identification and calibration

**Figure 16:**
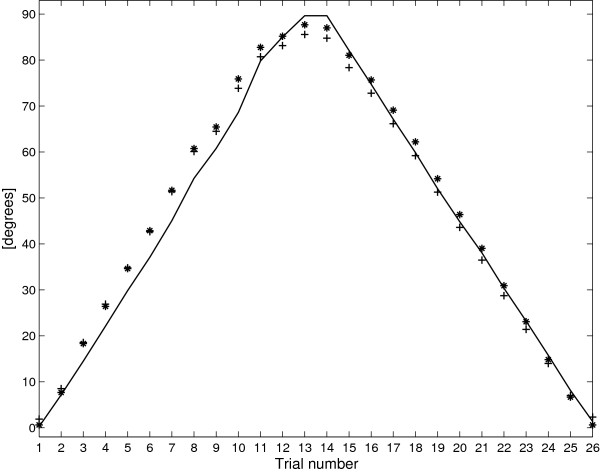
**Angle reconstruction through the calibrated KPF goniometer with calibration coefficients of (**23**) (expressed with +) and of (**31) (expressed with *) in comparison with biometrics electro-goniometer (solid line).

Figure 16 shows the results of the described identification methodology for an increasing/decreasing cycle. The output of equation (19), evaluated with coefficients (23) and (31), was compared with the Biometrics electrogoniometer. In terms of angular reconstruction, there was no significant difference between the two methods.

Given this result, the parameters can be reduced further. The knowledge that *a*=*a*_1_=−*a*_2_ and *q*_0_=*a*_3_ guarantees the correct functionality of the device. The two values required can be computed by calibrating the device in two points. In this way a rapid calibration procedure can be executed directly on the body after the goniometer has been integrated into the garment. After subtracting the initial value *q*_0_ computed as the average value of the resistance difference for *Δα*=0, it is sufficient to acquire the sensor output for a known angle (e.g. 90°) to compute *a*.

### Dynamic test

**Figure 17:**
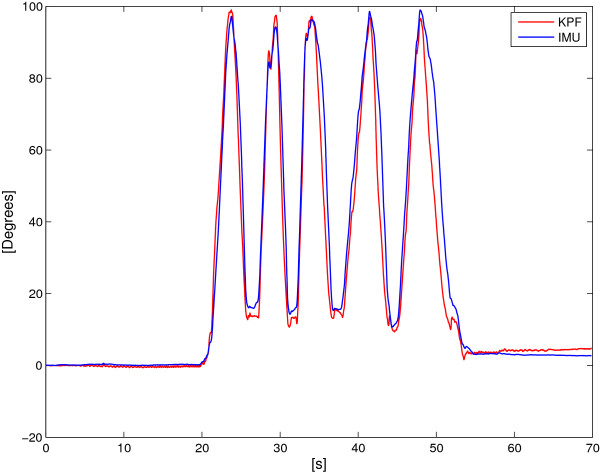
**Dynamic comparison between the calibrated KPF goniometer and the IMUs during a controlateral monopodalic standing task characterised by slow knee flexion-extension movements.** The red line represents the KPF goniometer output. The blue line is the knee flexion extension angle obtained by the two IMUs. Maximum error is 4.2°.

**Figure 18:**
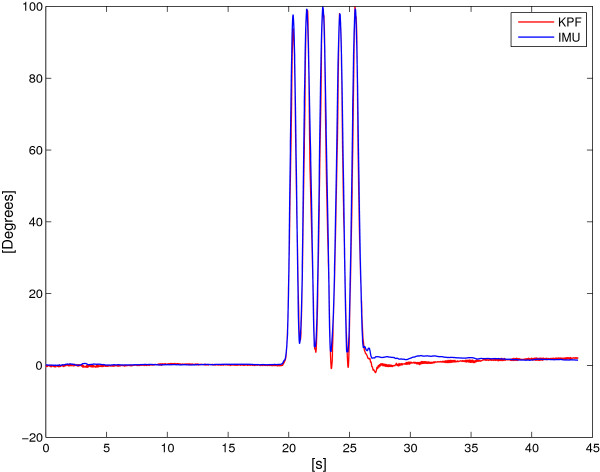
**Dynamic comparison between the calibrated KPF goniometer and the IMUs during a controlateral monopodalic standing task characterised by fast knee flexion-extension movements.** The red line represents the KPF goniometer output. The blue line is the knee flexion extension angle obtained by the two IMUs. Maximum error is 5°.

Figures 17 and 18 show two example comparisons between the DL KPF goniometer and IMUs during controlateral monopodalic standing tasks.

Both in the slow and fast knee flexion-extension, the double layer KPF goniometer performed well in angular measurements (maximum error of 5°) and managed to follow dynamic knee movements for compatible velocities with those of human movement.

To reinforce this first analysis, the statistical approach described in the Methods section was followed. Fixing a significance level as *η*=0.05, all the trials led to the same results, i.e. that there are no biases between the two measurement systems as reported in Table 1.

**Table 1:** Dynamic comparison between the KPF goniometer and IMUs in knee flexion-extension tasks

**Activity**	**X**	***σ***	**t**	***H***_**0**_
*Slow flexion*	0.06	0.28	-1.56	VERIFIED
*Normal flexion*	-0.4	0.33	0.93	VERIFIED
*Fast flexion*	0.08	0.40	0.20	VERIFIED

The KPF goniometer and IMUs angular measurements were compared in a set of different trials for different subjects. For each trial, statistics introduced in (33) and (34) were computed and a classical Student’s t-test was performed (*H*_*0*_-ipotesis, i.e. X is a zero-mean random variable).

## Discussion

Double layer KPF goniometers performed better than single layer sensors in terms of quasi-static angle reconstruction (5.3° vs. 8.3°).

Commercial electrogoniometers, such as those used in our quasi-static comparison with a declared accuracy of ±2°, are widely used in ambulatory measurements of the joint range of motion (ROM) and movement frequency/velocity/acceleration for both clinical and occupational evaluations [29-31]. Studies on goniometer accuracy have shown errors of a few degrees, with great dependence on the sensor positioning and on the cross talk between joints [32]. In [33] the evaluation of wrist ROM through electrogoniometers was performed with an error of between 2.2° and 6.2° over a flexion-extension range of 80°. Considering the widespread use of electrogoniometers, the reported errors are considered to be acceptable in clinical practice. The above reported KPF goniometer performances are slightly worse than those of commercial electrogoniometers, however they are still comparable with the ones accepted in goniometry applications.

KPF goniometers have also been compared with IMUs within dynamic trials. IMU-based joint kinematic estimations, widely described in [34], have a reconstruction accuracy that is lower than 3° for flexion-extension joint movements [35,36], making the good agreement of our dynamic test very promising.

The results reported in Figure 15 confirmed that the performance in extension (1.48°) is more accurate than the flexion performance (2.4°). It should also be noted that the angular error of the double layer sensor is mainly due to a hysteretic phenomenon pointed out in Figure 15 (i.e. maximum distance between the increasing and decreasing curve holds 10.4 *KΩ* corresponding to 10.9°), since the standard deviations (38) and (39) are consistently smaller than those of the double layer sensor (*σ*_*M* *a* *x*_).

**Figure 19:**
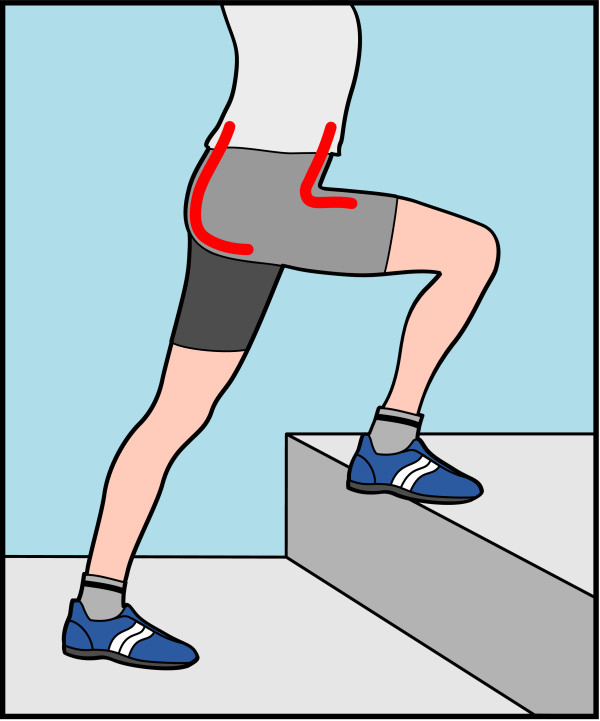
**Proposed configuration for hip flexion-extension detection.** Two goniometers are placed on the anterior and posterior sides of the pelvis girdle to improve the accuracy in terms of biomechanical reconstruction.

**Figure 20:**
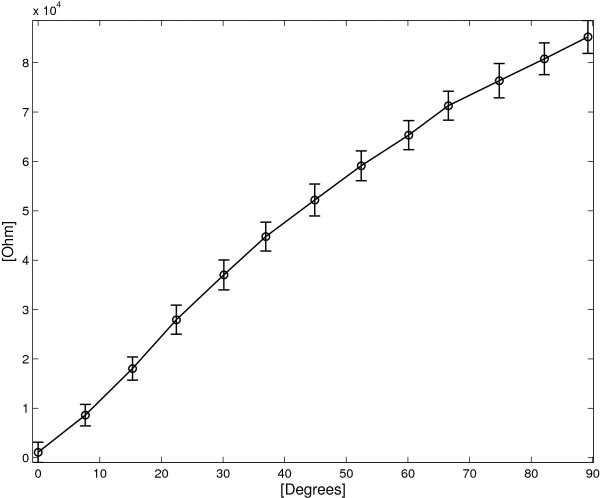
***ΔR***^***M***^** vs. angle for both flexion and extension cycles in the advanced configuration based on two KPF goniometers for hip flexion-extension detection.** The vertical bars represent two standard deviations in length.

Given this finding, the following approach was conceived in order to improve the overall accuracy in terms of biomechanical reconstruction with wearable goniometers. As a practical example we will now consider the problem of hip flexion-extension detection, although the proposed method could be generalized to many joint position reconstruction problems. Two different goniometers can be placed on the anterior and posterior sides of the pelvis girdle (e.g. corresponding to the proximal parts of the rectus femoralis and the gluteus maximus muscles), as shown in Figure 19. The hip flexion/extension movement is acquired by measuring the mean value *ΔR*^*M*^ between the anterior (*ΔR*^*A*^) and posterior sensor (*ΔR*^*P*^). This approach requires an on-body calibration, since the first angle between the abdominal region and the anterior part of the thigh and the second angle between the lumbar region and the surface of the hamstrings are not equivalent due to body structure differences. In this case an on-body calibration is needed to ensure that the two goniometers perform equivalent measurements (e.g. set the 0−*value* for each goniometer when the hip is completely extended). After this correction, the two sensor outputs are comparable and can be used to mutually integrate their information. In this approach, one of the two sensors will not return the effective angle between the two surfaces where it is applied, but the bias introduced will provide the re-calibrated sensor with the same behaviour as the other one in numerical terms. Thanks to this configuration, when one of the two sensors exhibits an increasing response, the other one will be decreasing. Our method reduces the error due to hysteresis: the new *ΔR*^*M*^ characteristic will be the mean of the increasing and decreasing responses and the overall standard deviation will be reduced, in the worse case, to the sum of the increasing and decreasing standard deviations. Figure 20 reports the *ΔR*^*M*^ characteristic, obtained by combining the increasing and decreasing responses.

The angular sensitivity was estimated in 960 *Ω*/° and is almost equal to that of a single goniometer. The maximum standard deviation is 3470 *Ω* which corresponds to an estimated angular standard deviation of 3.6° and represents a consistent improvement.

## Conclusions

We have developed a novel type of wearable goniometer based on KPF technology. In this paper, the working principle and the theoretical approach of single and double layer configurations have been described. On the basis of this theory, sensors were designed and produced on a fabric substrate. A calibration procedure that takes into account the dissimilarity between the two layers was proposed. Both single layer and double layer goniometers were tested in quasi-static conditions and compared with standard instrumentation. Double layer KPF goniometers performed better in terms of angle reconstruction compared to single layer ones (5.3° vs. 8.3° maximum error). In addition, double layer sensors are not sensitive to elongation and thus they are more suitable for applications in wearable motion detection. A preliminary dynamical evaluation showed how the quasi-static results could be extended in dynamic conditions. In addition, we demonstrated the overall sensor performance could be further improved through the fusion of two KPF goniometers per joint. Future work will focus on extending dynamic modeling and testing.

## Competing interests

The authors declare that they have no competing interests.

## Authors’ contributions

AT,FL defined the basic theory of sensors, the experiments to be performed and the experimental set-up, the data analysis and drafted the manuscript, supervised the research activity. GDM performed the experiments, developed the sensor electronics, drafted the sensor development, the experimental section of manuscript. NC performed the experiments, developed the sensor electronics, revised the manuscript. MP, RP produced the sensing material and drafted the sensor development section. DDR supervision and final revision. All authors read and approved the final manuscript.
